# Monitoring Autophagy at Cellular and Molecular Level in *Crassostrea gigas* During an Experimental Ostreid Herpesvirus 1 (OsHV-1) Infection

**DOI:** 10.3389/fcimb.2022.858311

**Published:** 2022-04-04

**Authors:** Sandy Picot, Nicole Faury, Camille Pelletier, Isabelle Arzul, Bruno Chollet, Lionel Dégremont, Tristan Renault, Benjamin Morga

**Affiliations:** ^1^Ifremer, ASIM, Adaptation Santé des invertébrés, La Tremblade, France; ^2^Ifremer, Département Ressources Biologiques et Environnement, La Tremblade, France

**Keywords:** autophagy, Pacific oyster (Crassostrea gigas), herpesvirus, innate immunity, invertebrate

## Abstract

Mortality outbreaks of young Pacific oysters, *Crassostrea gigas*, have seriously affected the oyster-farming economy in several countries around the world. Although the causes of these mortality outbreaks appear complex, a viral agent has been identified as the main factor: a herpesvirus called ostreid herpesvirus 1 (OsHV-1). Autophagy is an important degradation pathway involved in the response to several pathologies including viral diseases. In *C. gigas*, recent studies indicate that this pathway is conserved and functional in at least haemocytes and the mantle. Furthermore, an experimental infection in combination with compounds known to inhibit or induce autophagy in mammals revealed that autophagy is involved in the response to OsHV-1 infection. In light of these results, the aim of this study was to determine the role of autophagy in the response of the Pacific oyster to infection by virus OsHV-1. For this purpose, an experimental infection in combination with a modulator of autophagy was performed on Pacific oysters known to have intermediate susceptibility to OsHV-1 infection. In haemolymph and the mantle, the autophagy response was monitored by flow cytometry, western blotting, and real-time PCR. At the same time, viral infection was evaluated by quantifying viral DNA and RNA amounts by real-time PCR. Although the results showed activation of autophagy in haemolymph and the mantle 14 hours post infection (after viral replication was initiated), they were also indicative of different regulatory mechanisms of autophagy in the two tissues, thus supporting an important function of autophagy in the response to virus OsHV-1.

## Introduction

Increased hatchery production and livestock translocation contribute to the increasing occurrence and spread of infectious diseases among bivalves (Barbosa Solomieu et al., 2015). Recently, disease outbreaks have significantly affected farmed Pacific oysters in Europe and in other parts of the world (Barbosa Solomieu et al., 2015). Since 1990, mortality of spat of *Crassostrea gigas*, has been observed due to a virus called ostreid herpesvirus 1 (OsHV-1) in France and in other European and world regions (Hine et al., 1992; Nicolas et al., 1992; Renault et al., 1994a; Renault et al., 1994b; Friedman et al., 1997; Cherr and Friedman, 1998; Lynch et al., 2012; Peeler et al., 2012). This double-stranded DNA enveloped virus is currently the only known characterized member of the Malacoherpesviridae family, and its reference genotype was published in 2005 (Davison et al., 2005). In 2008, the emergence of a specific genotype of this virus called µVar was associated with mass mortality outbreaks among spat and juvenile *C. gigas* (Segarra et al., 2010). All the French oyster production areas were affected, and between 40% and 100% of Pacific oyster spat died.

Other studies have generally focused on the identification of antiviral compounds to expand the knowledge about the mechanisms underlying the resistance of the Pacific oyster to a viral infection (Bachère et al., 1990; Olicard et al., 2005; Renault et al., 2011; Green et al., 2014). The recent publication of *Crassostrea gigas* genome (Zhang et al., 2012) has allowed identifying several pathways involved in immune-system mechanisms (He et al., 2015; Moreau et al., 2015; Rosani et al., 2015). These studies suggest that several mammal innate immune pathways exist in this specie. It has been suggested that *C. gigas* can control a viral infection by means of the RNA interference (RNAi) pathway, an interferon-like pathway, apoptosis, and *via* autophagy (Zhang et al., 2011; Green and Montagnani, 2013; Segarra et al., 2014a; Segarra et al., 2014c; Green et al., 2015; He et al., 2015; Moreau et al., 2015; Martenot et al., 2017).

Macroautophagy, which is more commonly simply called autophagy, is a pathway widely conserved among eukaryotes. This process involves engulfment of a portion of the cytoplasm with components of the cell (from proteins to whole organelles) for their degradation by fusion with lysosomes (Levine and Deretic, 2007). Autophagy participates in key cellular processes including cellular homeostasis, starvation adaptation, cell death, and immune response to pathogens (Klionsky and Emr, 2000; Mizushima, 2005; Deretic, 2006; Schmid and Münz, 2007). This cellular mechanism can block the replication of (or infection by) different pathogens including viruses, bacteria, and parasites.

In *C. gigas*, autophagy has previously been characterized in the mantle and haemocytes (Picot et al., 2020). In these two oyster compartments, autophagy has been successfully modulated after exposing oysters to molecules well known to modulate the autophagy pathway in mammals (Moreau et al., 2015; Picot et al., 2019). The mantle has been reported to be a target organ of OsHV-1 (Renault et al., 1994a; Renault et al., 2001; Schikorski et al., 2011a; Segarra et al., 2016). The presence of viral mRNA is detected earlier in the mantle compared to the other organs (Segarra et al., 2014b; Segarra et al., 2014c). Haemocytes are the principal effectors of the oyster immune system (Allam and Raftos, 2015). It has been suggested that haemocytes are carrier cells responsible for the transport of OsHV-1 to target organs during the first stages of viral infection (Schikorski et al., 2011a; Segarra et al., 2016; Morga et al., 2017). One study showed that 20 h post infection (hpi), the autophagy pathway is implying in the presence of virus OsHV-1 in the mantle of *C. gigas* (Moreau et al., 2015).

To clarify the role of autophagy in the response of the Pacific oyster to OsHV-1 infection, experimental infections were carried out using respectively a known inhibitor of autophagy (NH_4_Cl). Both autophagy and the development of the virus were monitored concurrently in the mantle and haemolymph. Autophagy was measured using cellular (flow cytometry and western blotting) and complementary molecular approaches (real-time PCR) previously developed and applied by (Picot et al., 2019; Picot et al., 2020). The viral DNA load and expression of three viral genes were monitored by real-time PCR during the experimental infection. Thanks to an integrated approach, this study has revealed that autophagy is activated in the mantle and haemolymph of *C. gigas* after the initiation of OsHV-1 replication. Interestingly different autophagy regulatory mechanisms seem to occur in the two tissues in response to OsHV-1.

## Materials and Methods

### Oyster Production

*Crassostrea gigas* spat were produced at the Ifremer facilities in La Tremblade (Charente-Maritime, France) from one family. This family was selected for its intermediate susceptibility to the viral infection when tested under experimental conditions as described by Segarra et al. (2014b). Spawn occurred in May 2016, and larval and spat cultures were performed as described by Dégremont et al. (2005) and Azéma et al. (2017). All growth steps involved filtered and UV-treated seawater to prevent contamination with pathogens naturally present in the environment, including OsHV-1 and *Vibrio aestuarianus*.

Prior to the experiment, spat were acclimated *via* a constant flow of filtered and UV-treated seawater enriched in phytoplankton (*Skeletonema costatum*, *Isochrysis galbana*, and *Tetraselmis suecica*) in 120 L tanks at 19°C for at least 2 weeks.

### Experimental Design Including Pharmacological Agent and Virus OsHV-1

Seven hundred and fifty oysters (3-4cm) were chloride induced a myorelaxing for 4 h in a solution containing magnesium chloride (MgCl_2_, 50 g/L) in seawater mixed with distilled water (1:4, v/v) (Schikorski et al., 2011b). Four conditions were tested, each replicated by 12 tanks, and each tank containing 15 oysters: oysters either injected with 100 µL of an OsHV-1 suspension at 1 × 10^4^ copies of viral DNA/µL or injected with seawater, which were subsequently either kept in seawater or kept in seawater supplemented with NH_4_Cl at 1 mM). Two tanks of each condition were sampled at 6, 10, 14, 18, 24, and 30 h post infection. At T0 (time before oysters were incubated under the different tested conditions), two pools of 15 oysters were sampled to determine the basal level of autophagy in the mantle and haemolymph. At each sampling time and for each condition, pieces of mantle were collected from six oysters to quantify viral DNA and measure viral and autophagy gene expression, and western blotting. In parallel, haemolymph was withdrawn from the adductor muscle of oysters with a 1 mL syringe equipped with a needle (0.6 × 32 mm) as described by Picot et al. (2019). The haemolymph of the 15 oysters in each tank was pooled for viral DNA quantification, analysis of viral and autophagy genes expression and flow cytometry.

Survival was monitored for 7 days after injection (three additional tanks of 15 oysters per condition). Percentages of cumulative survival were determined daily for the different conditions. Dead oysters were removed from tanks in the course of the experiment.

### DNA Extraction

Total DNA was extracted from the mantle or haemolymph using the QiaAmp DNA Mini Kit (Qiagen), according to the manufacturer’s protocol. Elution was performed in 200 µL (for mantle extraction) and 50 µL (for haemolymph extraction) of AE buffer provided in the kit. The DNA quality and quantity were determined on a NanoDrop 2000 instrument (Thermo Scientific, http://www.nanodrop.com).

### OsHV-1 DNA Quantification by Real-Time PCR

OsHV-1 DNA quantification was estimated by real-time PCR in duplicate according to Pepin et al. (2008) on a Mx3000 Thermocycler sequence detector (Agilent). Amplification reactions were carried out in a total volume of 20 µL. Each well contained 5 µL of genomic DNA (5 ng/mL), 10 µL of Brillant III Ultra-Fast SYBR Green Master Mix (Agilent), 2 µL of each primer (5.5 µM: OsHV-1 DPFor 5′-ATTGATGATGTGGATAATCTGTG-3′, 5.5 µM OsHV-1 DPRev 5′-GGTAAATACCATTGGTCTTGTTCC-3′) (Webb et al., 2007), and 1 µL of distilled water. Real-time PCR cycling conditions were as follows: 3 min at 95°C, followed by 40 cycles of amplification at 95°C for 5 s and 60°C for 20 s. The results were expressed as log_10_ of virus DNA copy numbers per nanogram of total DNA.

### Total RNA Extraction and cDNA Synthesis

From each tissue, total RNA was extracted with the TRIzol™ Reagent (Ambion) according to the manufacturer’s recommendations. Total RNA was treated with TURBO™ DNase (Invitrogen) to remove genomic DNA. The quality and quantity of the RNA were determined on the NanoDrop 2000 (Thermo Scientific). Mock reverse transcription was performed after each DNase treatment to verify the absence of oyster and/or viral genomic DNA. First-strand cDNA synthesis was carried out by means of SuperScript^®^ III Reverse Transcriptase (Invitrogen) with 500 ng of the treated RNA.

### Expression of Viral Genes

Real-time PCR was carried out to monitor the expression of three viral genes (ORFs 80, 87, and 99). These three ORFs were selected based on their protein functions and early expression during the viral infection (Davison et al., 2005; Segarra et al., 2014b; Morga et al., 2017). ORF 80 encodes a putative membrane protein and ORFs 87 and 99 apoptosis inhibitors. The expression of the three viral genes was evaluated following the protocol described by Segarra et al. (2014a) with 5 μL of cDNA dilution (1/30) instead of genomic DNA. Normalized relative viral gene expression levels were calculated for each sample with the formula: Delta C_t_ = C_t_ ORF − C_t_ Elongation factor 1alpha (EF1-α). The gene expression level (Delta C_t_) of the initial array data was transformed as follows: [1 − (Delta C_t_/C_t_ EF1-α)]/100. C_t_ (threshold cycle) corresponds to the PCR cycle number.

### Expression of Oyster Immunity Genes

Moreover, the relative expression of seven immunity genes in *C. gigas* spat was analyzed during the OsHV-1 experimental infection at T0, 6, 10, 14, 18, 24, and 30 hpi. The relative quantification value (ratio R) was calculated by the method described by Pfaffl (2001):


$$R=\left[\left(E_{target}\right)\cdot^{\Delta CTtarget\left(control-sample\right)}\right]/\left[\left(E_{ref}\right)\cdot^{\Delta CTref\left(control-sample\right)}\right]$$

The efficiency of each primer pair was determined by constructing a standard curve from serial dilutions (**Table 1**). These five genes of the Pacific oyster were (i) sequestosome 1 (*SQSTM1*), (ii) microtubule-associated protein 1A/1B light chain 3A (*MAP1LC3A*), (iii) beclin-1 (*BECN1*), (iv) serine/threonine protein kinase ULK2 (*ULK2*), and (v) autophagy-related protein 7 (*ATG7*; **Table 1**). Host gene expression was normalized to EF1-α, because no significant differences in C_t_ values were observed for this housekeeping gene among several conditions in the course of the experiment. The calibrator in this experiment was individuals sampled at T0.

**Table 1:** List of primer for viral ORF and C. gigas autophagy genes expressions.

Categories	Gene name/ORFs	Forward	Reverse	Effeciency	Protein
**Autophagy genes**	BECN1	AATGCTGCTTGGGGTCAGA	CGGAATCCACCAGACCCATA	102.2	PI3KC3 complex
	ULK2	CTGACTTTGGCTTTGCTCGT	TTTGAGCTGTTGAGGGGTCT	103.9	Atg1/ULK1 complex
	MAP1LC3A	CCGATGCTTGACAAGACCAA	CCGTCCTCGTCTTTCTCCTG	98.2	LC3 conjugation system
	P62/SQQTM1	AGGGAATGAGAAGGCCGAAA	CCTCAAGCAACTCCTCTCCA	96.5	Delivers ubiquitinated cargoes for autophagic degredation
	ATG7	CGCCCCTTGTAAACAAAATG	ATTCTGCAAGGCATTCCAAC	104.8	LC3 and ATG12 conjugation systems
**OsHV-1 genes**	ORF80	AAGAGGATTTGGGTGCACAG	TTGCATCCCAGGATTATCAG	98.5	Membrane protein
	ORF87	CACAGACGACATTTCCCCAAA	AAAGCTCGTTCCCACATTGGT	98.7	Inhibitor of apoptosis protein
	ORF99	GGTGGAGGTGGCTGTTGAAA	CCGACTGACAACCCATGGAC	96.3	Inhibitor of apoptosis protein

### Flow Cytometry

Before autophagic activity was investigated in haemocytes, haemocyte mortality was determined. As described by Gagnaire (2005), haemocyte mortality was measured in 200 μL of a cell suspension sampled from each condition (two replicates) and at each sampling time point. The cells were incubated in the dark for 30 min at 4°C with 10 μL of propidium iodide (PI, 1 mg/mL; Thermo Fisher Scientific, cat. # P3566).

Then, percentages of haemocytes with autophagic activity were quantified with the commercial Cyto-ID^®^ autophagy detection kit (ENZO Life Sciences, ENZ-51031-K200) as described by Picot et al. (2020).

For each sample, 5000 events were acquired on an EPICS XL 4 cytometer (Beckman Coulter, USA). Size discrimination was implemented to ensure that small particles or bacteria were not counted, so that only haemocytes were taken into account when cell activity was measured. The results were depicted as cell cytograms and reported as log scale fluorescence levels of each marker tested. The results were expressed as differences between the percentage of haemocytes that positively presented autophagosomes for each condition and the percentage of haemocytes labeled in the artificial seawater condition at each sampling time point. Flow cytometry data were analyzed in Flowing software 2.5.1 (University of Turku, Finland).

### Western Blot

Pieces of mantle were collected from Pacific oysters (20 to 25 mg). Mantle protein extraction and western blot were performed as reported by Picot et al. (2020). Thirty micrograms of each pool of the mantle protein extracted was loaded onto an SDS polyacrylamide gel (Bio-Rad). Primary antibodies against Actin (A4700, Sigma-Aldrich), SQSTM1 (P0067, Sigma-Aldrich) were respectively diluted at 1/1000, 1/500, and 1/6000.

MAP1LC3-II/actin and SQSTM1/actin ratios were calculated based on densitometry analysis of the bands in the ImageJ software (v. 1.51q). Each sample was normalized to actin and calibrated in comparison with the control condition for each experiment.

### Data Analysis

All analyses were conducted in the R studio software (version 3.3.2). First, normality of all the datasets was tested by the Shapiro–Wilk test, and homogeneity of variances was assumed because of the results of Bartlett’s test.

Kaplan–Meier survival curves and the logrank test were used to characterize and compare survival between oyster conditions (packages survival, v2.39-5, and survminer, v. 0.4.3).

OsHV-1 DNA amounts were compared between groups “virus” and “virus+NH_4_Cl” for each tissue by the Kruskal–Wallis test (package PMCMR, v. 4.1). In haemolymph, the sampling time points were subdivided into two categories. The first category consisted of the early sampling time points (6, 10, and 14 hpi), and the second of the late sampling time points (18, 24, and 30 hpi). For the mantle, results of each sampling time point were tested separately. Scatterplots and trend curves were built with package ggplot2 (version 2.2.1).

Relative expression of oyster and viral genes are presented as scatterplots with trend curves (package ggplot2, v 2.2.1). The Kruskal–Wallis test was carried out to determine whether significant differences exist between experimental treatments at different sampling time points. In haemolymph, the difference was tested at early (6, 10, and 14 hpi) and late time points (18, 24, and 30 hpi) of the experimental infection. For the mantle, results of each sampling time point were tested separately.

Flow cytometry data were compared between the conditions tested and the artificial seawater condition at early (6, 10, and 14 hpi) and late (18, 24, and 30 hpi) time points of the experimental infection by Student’s *t* test. Scatterplots and trend curves were constructed using package ggplot2.

Western blot data were compared by the Mann–Whitney test between the conditions tested and the artificial seawater group at early and late time points of the experimental infection. Bar plots were built by means of package ggplot2.

## Results

### Mortality

**Figure 1:**
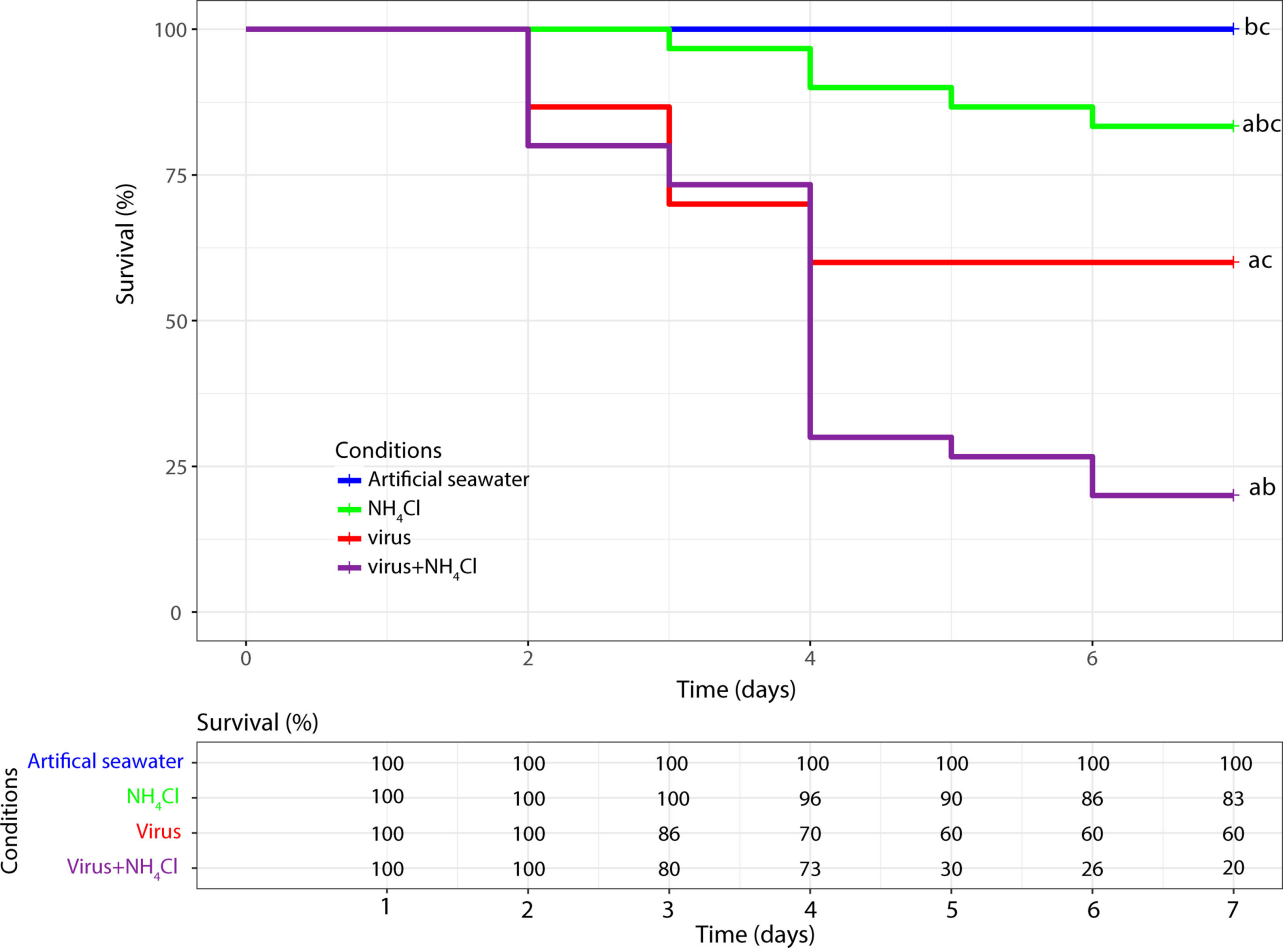
Kaplan-Meier survival curves of the pacific oyster *Crassostrea gigas* injected with the virus OsHV-1, exposed to NH_4_Cl or injected with OsHV-1 and exposed to NH_4_Cl. The control condition corresponds to pacific oysters injected with artificial sea water. In each condition the mortality was monitored on n=30 oysters. a: significant difference with the artificial seawater condition (p < 0.05). b: significant difference with the virus condition (p < 0.05). c: significant difference with the virus+NH_4_Cl condition (p < 0.05).

To assess the effects of OsHV-1 and NH_4_Cl on Pacific oyster spat, survival was monitored for 7 days post infection (dpi). No oyster mortality was detected in the artificial seawater group (**Figure 1**). In the NH_4_Cl group, the mean survival rate was 83% at 7 days post exposure. The presence of virus OsHV-1 and bacterium *V. aestuarianus* was investigated by real-time PCR in dead animals. The results did not allow us to detect the bacterium or the virus in any dead animal. The mean survival rates in the virus group (60%) and virus+NH_4_Cl group (20%) were significantly different from the results obtained for the seawater group at 7 dpi (p ≤ 0.05). At the same time, significant differences in the mean survival rate were observed between the NH_4_Cl and virus+NH_4_Cl groups (p ≤ 0.05) and the virus and virus+NH_4_Cl groups (p ≤ 0.05).

### Detection of OsHV-1 DNA and RNA in the Mantle and the Haemolymph of *C. gigas*

**Figure 2:**
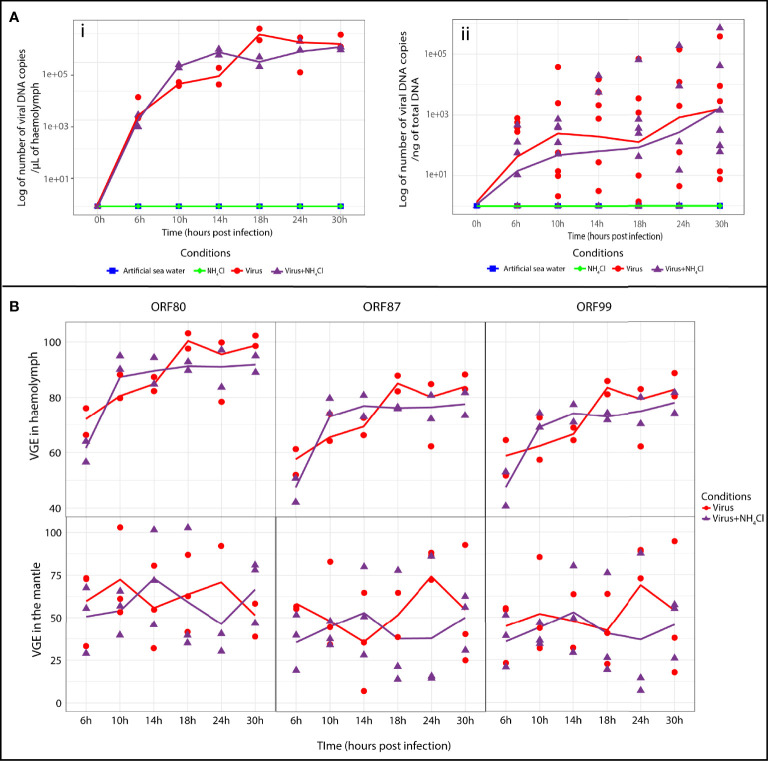
Detection of OsHV-1 DNA and RNA in haemolymph and mantle of *Crassostrea gigas*. **(A)** Viral DNA amounts detected by real time PCR in Pacific oysters of the four different tested conditions (artificial seawater, NH_4_Cl, virus and virus+NH_4_Cl) after injection by OsHV-1 (i) in haemolymph (n=2 pools of 15 animals) and (ii) in the mantle. (n=6 animals) The line represents the trend curve of viral DNA amount in the virus condition (red line) and the virus+NH_4_Cl condition (purple line). No significant difference was observed between the virus and virus+NH_4_Cl conditions in the mantle and haemolymph. **(B)** Relative genes expressions of ORF 80, ORF 87 and ORF 99 estimated by RT-PCR at different time of exposure for the virus and virus+NH_4_Cl condition in haemolymph (n= 2 pools of 15 animals) and mantle (n=3 animals). The line represents the trend curve of the expression of each gene in the virus condition (red line) and the virus+NH_4_Cl condition (purple line). No significant difference was observed between the virus and virus+NH_4_Cl condition in haemolymph and the mantle of the Pacific oyster. VGE, viral gene expression.

Viral DNA detection was conducted in the haemolymph and mantle of Pacific oysters during the process of infection (**Figure 2A**). Viral DNA and RNA amounts were monitored at each sampling time point (T0, 6, 10, 14, 18, 24, and 30 hpi).

In haemolymph and the mantle, no viral DNA was detected in artificial seawater and NH_4_Cl groups (**Figure 2A**). OsHV-1 DNA could be detected as early as 6 hpi in both tissues tested in the virus group and virus+NH_4_Cl group. In haemolymph (**Figure 2A**), the viral DNA amount increased from 6 to 18 hpi in the virus group (1.03 × 10^6^ ± 6.21 × 10^5^ viral DNA copies/µL of haemolymph) to 24 hpi in the virus+NH_4_Cl group (4.02 × 10^5^ ± 1.91 × 10^5^ viral DNA copies/µL of haemolymph). After that, the viral DNA amount tended to stay stable until the end of the experiment in the two groups. No significant difference was observed between the virus group and virus+NH_4_Cl group at early (6 to 14 hpi) and late (18 to 30 hpi) time points of the experimental infection in haemolymph. In the mantle (**Figure 2A**), viral DNA amounts increased from 6 until 30 hpi in virus and virus+NH_4_Cl groups. The maximal viral DNA amounts were detected at 30 hpi in the virus group (6.57 × 10^4^ ± 1.54 × 10^5^ viral DNA copies/µL of haemolymph) and in the virus+NH_4_Cl group (1.26 × 10^5^ ± 2.89 × 10^5^ viral DNA copies/µL of haemolymph, respectively). No significant difference was observed between the virus group and virus+NH_4_Cl group at any sampling time point in the mantle.

The expression of three viral ORFs was monitored by real-time PCR (**Figure 2B**) to estimate viral replication in the haemolymph and mantle under the different conditions at each sampling time point. In the mantle and haemolymph, the first detection of viral RNA of the three genes was observed at 6 hpi in the virus group and virus+NH_4_Cl group (**Figure 2B**). In haemolymph, the viral RNA amount increased exponentially at the beginning of the experiment and tended to stay stable until the end of the experiment in the virus group and virus+NH_4_Cl group (**Figure 2B**). In the mantle, the viral transcripts of the three ORFs were detected from 6 until 30 hpi, but their expression manifested high variation among individuals (**Figure 2B**). No significant differences in the relative expression of each ORF was thus detected between the two groups in the course of the experiment in haemolymph and the mantle.

### Monitoring of Haemocyte Mortality in Oyster Haemolymph

Prior to monitoring the autophagic activity by flow cytometry, haemocyte mortality was evaluated with PI. In all the tested groups at all sampling time points, the mean cell mortality never exceeded 11.9%.

### Monitoring of Autophagic Activity in Oysters Exposed to OsHV-1

**Figure 3:**
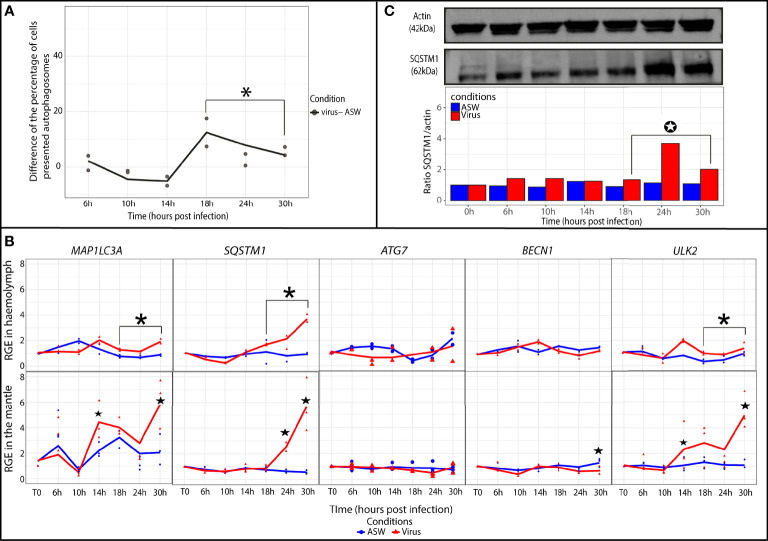
Modulation of the autophagy in *Crassostrea gigas* exposed to OsHV-1. **(A)** Monitoring of autophagy in haemocytes of *C. gigas* by flow cytometry. Scatterplot of the difference of the percentage of haemocytes presented autophagosomes between the seawater condition (ASW) and the virus condition from 6 to 30 hpi (n=2 pools of 15 animals). The line represents the trend curve of the difference of percentage of cells between the two conditions. *Significant increase of the difference of the percentage of cells presented autophagosomes between the virus and seawater condition (p < 0.05). **(B)** Relative gene expression of key autophagy genes in haemolymph and the mantle of the Pacific oysters, *C. gigas*, in ASW condition (blue) and virus condition (red) at each sampling time point (T0, 6, 10, 14, 18, 24 and 30 hpi) detected by real time PCR (haemolymph: n=2 pools of 15 animals; mantle: n=3 animals). The line represents the trend curve of the expression of each gene in the virus condition (red) and the ASW condition (blue). Significant difference between the virus and ASW condition at early (6-14 hpi) or late time points (18-30 hpi) of the experimental infection (p < 0.05). Significant difference between the virus and ASW conditions of the same time point (p < 0.05). RGE, relative gene expression. **(C)** Detection of the autophagy protein SQSTM1 in the mantle of *C. gigas* during the kinetic of infection by OsHV-1 in the virus condition at each sampling time point (for each time point n=1 pool of 3 animals). Significant differences between the virus and ASW condition at early (6-14 hpi) or late time points (18-30 hpi) of the experimental infection (p < 0.05).

In the oyster haemolymph from the virus group, the autophagic activity was determined by calculating the difference in the percentages of cells containing autophagosomes between the virus group and artificial seawater group (**Figure 3A**). At late (18 to 30 hpi) time points of the experimental infection, the difference significantly increased and stayed positive in comparison with early (6 to 14 hpi) time points of the experimental infection (p ≤ 0.05). These results indicated that the percentage of cells containing autophagosomes was higher in the virus group between 18 and 30 hpi. The relative expression of five autophagy genes was monitored in the oyster haemolymph from the virus group (**Figure 3B**). The results revealed that the relative expression of *ULK2*, *SQSTM1*, and *MAP1LC3A* was significantly higher in the virus group in comparison with the artificial seawater group at later time points of the experimental infection (18 to 30 hpi; p ≤ 0.05).

In the mantle, *MAP1LC3A* and *ULK2* relative expression levels were significantly higher at two time points (14 and 30 hpi; p ≤ 0.05) in the virus group (**Figure 3B**). The relative expression of *SQSTM1* significantly increased from 24 to 30 hpi (p ≤ 0.05). Relative expression of *BECN1* significantly increased at 30 hpi (p ≤ 0.05). *ATG7* relative expression did not seem to vary during the experiment. At the protein level, the SQSTM1/actin ratio was determined by western blotting to follow the protein quantity of SQSTM1 in the mantle of *C. gigas* (**Figure 3C**). The results indicated that the SQSTM1 protein quantity significantly increased in the virus group relative to the seawater group at the later time points of the experiment (18 to 30 hpi).

### Monitoring of Autophagic Activity in Oysters Exposed to NH_4_Cl

**Figure 4:**
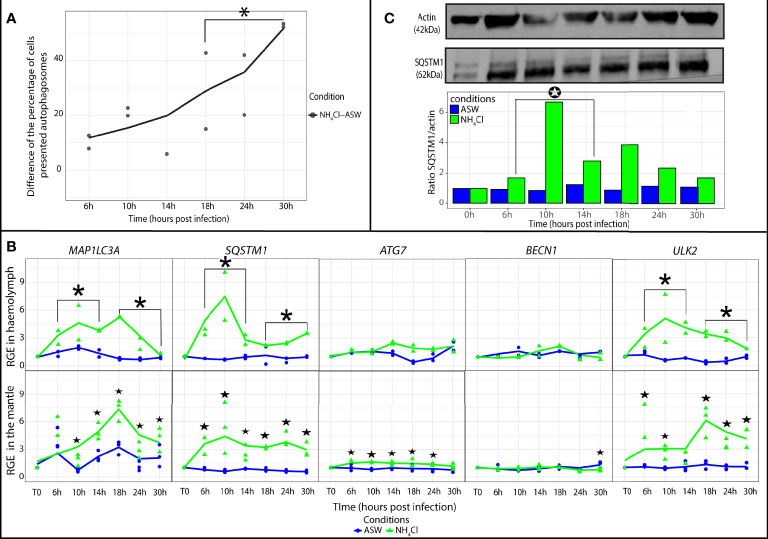
Modulation of autophagy in *Crassostrea gigas* exposed to NH_4_Cl. **(A)** Monitoring of autophagy in haemocytes of *C. gigas* by flow cytometry. Scatterplot of the difference of the percentage of haemocytes presented autophagosomes between the seawater condition (ASW) and the NH_4_Cl condition from 6 to 30 hpi (n=2 pools of 15 animals). The line represents the trend curve of the difference of percentage of cells between the two conditions. *Significant increase of the difference of the percentage of cells presented autophagosomes between the NH_4_Cl and ASW condition between 18 to 30hpi (p < 0.05). **(B)** Relative gene expression of key autophagy genes in haemolymph and the mantle of the Pacific oysters, *C. gigas*, in ASW condition (blue) and NH_4_Cl condition (green) at each sampling time point (T0, 6, 10, 14, 18, 24 and 30 hpi) detected by real time PCR (haemolymph: n=2 pools of 15 animals; mantle: n=3 animals). The line represents the trend curve of the expression of each gene in the NH_4_Cl condition (green) and the ASW condition (blue). Significant difference between the NH_4_Cl and ASW conditions at early (6-14 hpi) or late time points (18-30 hpi) of the experimental infection (p < 0.05). Significant difference between the NH_4_Cl and ASW conditions of the same time point (p < 0.05). RGE, relative gene expression. **(C)** Detection of the autophagy protein SQSTM1 in the mantle of *C. gigas* during the kinetic of exposition to NH_4_Cl at each sampling time point (for each time point n=1 pool of 3 animals). Significant differences between the NH_4_Cl and ASW condition at early (6-14 hpi) or late time points (18-30 hpi) of the experimental infection (p < 0.05).

The monitoring of autophagic activity by flow cytometry revealed a significant increase in the percentage of cells containing autophagosomes in the NH_4_Cl group in comparison with the artificial seawater group at late time points [18 to 30 h post exposure (hpe)] of the experimental exposure to NH_4_Cl (p ≤ 0.05; **Figure 4A**). On the other hand, in the NH_4_Cl group, the relative expression of *MAP1LC3A*, *ULK2*, and *SQSTM1* was higher at early (6 to 14 hpe) and late time points (18 to 30 hpe) of the experimental infection in comparison with the artificial seawater group (p ≤ 0.05). *ATG7* and *BECN1* expression showed no significant difference between the NH_4_Cl group and artificial seawater group.

In the mantle, genes *MAP1LC3A* and *ULK2* were significantly upregulated respectively from 10 to 30 hpe and from 6 to 30 hpe (p ≤ 0.05) in the NH_4_Cl group (**Figure 4B**). *SQSTM1* was significantly upregulated from 10 to 30 hpe (p ≤ 0.05). The relative expression of *ATG7* and *BECN1* stayed weak in comparison with their expression levels in the seawater group. Nevertheless, *ATG7* was significantly upregulated from 6 to 24 hpe, and *BECN1* was upregulated at 30 hpe (p ≤ 0.05) in the presence of NH_4_Cl. Moreover, we observed an increase in the protein SQSTM1 amount by western blot analysis. In fact, the results indicated that the SQSTM1 protein quantity was significantly higher in the NH_4_Cl group than the artificial seawater group during early (6 to 14 hpe) time points of the experimental infection (p ≤ 0.05; **Figure 4C**).

### Monitoring of Autophagic Activity in Oysters Exposed to Virus+NH_4_Cl

**Figure 5:**
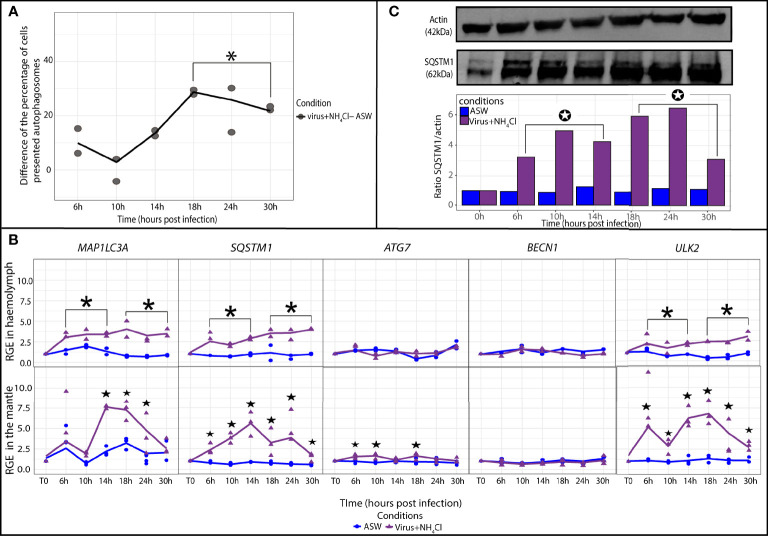
Modulation of autophagy in *Crassostrea gigas* exposed to virus+NH_4_Cl. **(A)** Monitoring of autophagy in haemocytes of *C. gigas* by flow cytometry. Scatterplot of the difference of the percentage of haemocytes with autophagosomes between the seawater condition (ASW) and the virus+NH_4_Cl condition from 6 to 30 hpi (n=2 pools of 15 animals). The line represents the trend curve of the difference of percentage of cells between the two conditions. *Significant increase of the difference of the percentage of cells presented autophagosomes between the virus+NH_4_Cl and ASW condition between 18 to 30hpi (p < 0.05). **(B)** Relative gene expression of key autophagy genes in haemolymph and the mantle of the Pacific oysters, *C. gigas*, in seawater condition (blue) and the virus+NH_4_Cl condition (purple) at each sampling time point (T0, 6, 10, 14, 18, 24 and 30 hpi) detected by real time PCR (haemolymph: n=2 pools of 15 animals; mantle: n=3 animals). RGE, relative gene expression. The line represents the trend curve of the expression of each gene in the virus+NH_4_Cl condition (purple) and the ASW condition (blue). Significant difference between the virus+NH_4_Cl and ASW conditions at early (6-14 hpi) or late time points (18-30 hpi) of the experimental infection (p < 0.05). Significant difference between the virus+NH_4_Cl and ASW conditions of the same time point (p < 0.05). **(C)** Detection of the autophagy protein SQSTM1 in the mantle of *C, gigas* during the kinetic of infection by OsHV-1 in the virus+NH_4_Cl condition at each sampling time point (for each time point n=1 pool of 3 animals). Significant differences between the virus+NH_4_Cl and ASW condition at early (6-14 hpi) or late time points (18-30 hpi) of the experimental infection (p < 0.05).

The monitoring of the autophagic activity by flow cytometry suggested that the percentage of cells containing autophagosomes was significantly higher in the virus+NH_4_Cl group than in the artificial seawater group at late (18 to 30 hpi) time points of the experimental infection (p ≤ 0.05; **Figure 5A**). At the molecular level, the relative expression of genes *MAP1LC3A*, *SQSTM1*, and *ULK2* was significantly upregulated at early (6 to 14 hpi) and late (18 to 30 hpi) time points in the virus+NH_4_Cl group in comparison with the artificial seawater group (p ≤ 0.05; **Figure 5B**). *BECN1* and *ATG7* expression showed no significant differences between the two tested groups.

In the mantle, relative expression of *MAP1LC3A* was significantly higher from 14 to 24 hpi in the virus+NH_4_Cl group in comparison with the seawater group (p ≤ 0.05; **Figure 5B**). *ULK2* was significantly upregulated from 6 to 30 hpi (p ≤ 0.05). *SQSTM1* was significantly upregulated in comparison with the seawater group from 6 to 30 hpi (p ≤ 0.05). *ATG7* was statistically but weakly upregulated at 6, 10, and 18 hpi (p ≤ 0.05). Relative expression of *BECN1* did not vary during the experiment. The protein ratio SQSTM1/actin confirmed the increase in the quantity of the SQSTM1 protein in the virus+NH_4_Cl group relative to the seawater group at early (6 to 14 hpi) and late (18 to 30 hpi) time points of the experiment (p ≤ 0.05; **Figure 5C**).

## Discussion

*C. gigas* is the most important aquaculture farming resource in France. This specie is widely cultivated due to its good growth capacity and resistance to environmental factors (FAO, 2018b; FAO, 2018a). Nonetheless, since 1990, virus OsHV-1 has been responsible for mortality events among Pacific oyster spat (Nicolas et al., 1992; Renault et al., 1994a; Renault et al., 1994b). Despite the impact of the mortality caused by this virus on the aquaculture economy, few countermeasures are available. The innate immunity mechanisms of *C. gigas* involved in the response to OsHV-1 need to be documented more thoroughly. Recently, autophagy, one of the innate immunity pathways of the Pacific oyster, was investigated in the mantle of *C. gigas* and was demonstrated to participate in the response to OsHV-1 infection (Moreau et al., 2015). In that study, autophagy was investigated in a single tissue of the Pacific oyster, the mantle, and at a single time point during the viral infection.

In the present study, we investigated the role played by autophagy in *C. gigas* during OsHV-1 infection using an integrated approach. An experimental infection was carried out in combination with a known inhibitor of the autophagy pathway, ammonium chloride (NH_4_Cl). During this experiment, autophagy kinetic was monitored in the mantle and haemolymph by different cellular (flow cytometry, western blot) and molecular (real-time PCR) approaches.

The first experimental infection was performed by intramuscular injection of a viral suspension into *C. gigas* spat. Oyster survival was monitored for 7 dpi. Higher mortality rates were observed in oysters injected with the virus and exposed to NH_4_Cl and to a lesser extent in oysters injected only with the virus. Similar results were obtained by Moreau et al. (2015). Nevertheless, these authors did not detect mortality in oysters exposed to NH_4_Cl alone. In our experiment, a low mortality rate (17%) was observed among the oysters exposed to NH_4_Cl alone. Because OsHV-1 or *V. aestuarianus* DNA were not detected, these deaths appeared to be unrelated to these pathogens generally responsible for *C. gigas* mortality. On the other hand, the presence of other pathogens that may kill *C. giga*s was not investigated here. Moreover, the animals used in the experiment were mature. *C. gigas* is highly sensitive to changes in biotic and abiotic factors during gametogenesis (Berthelin et al., 2000; Li et al., 2007; Enríquez-Díaz et al., 2008). Perhaps sexually mature animals are more susceptible to NH_4_Cl exposure than immature oysters. Finally, exploration of autophagy in relation to viral infection was performed before first deaths were observed.

First, the modulation of autophagy was investigated in Pacific oysters exposed to NH_4_Cl. This reagent was employed in the experiment owing to its capacity to inhibit autophagy, namely, specific suppression of autophagosome degradation (Sharifi et al., 2015; Klionsky et al., 2016). In haemolymph and the mantle, our results uncovered modulation of autophagy genes starting from 6 hpe. NH_4_Cl induced a modulation of the autophagy genes. Moreover, the percentage of cells containing autophagosomes and expressing protein SQSTM1 significantly increased respectively since 18 and 6 hpe. SQSTM1 is a protein required for the formation and degradation of polyubiquitin-containing bodies *via* autophagy (Pankiv et al., 2007). This protein is a marker used to study autophagy flux (Bjørkøy et al., 2009). Our results indicated that NH_4_Cl induced accumulation of the SQSTM1 protein in the mantle. In the haemolymph, accumulation of autophagosomes was observed through an increase in the percentage of haemocytes containing autophagosomes. This analysis indicates that in the two tissues, autophagy was functional and was inhibited by NH_4_Cl. Similar results were already obtained in the haemocytes and mantle of *C. gigas* by means of the same reagent (Moreau et al., 2015; Picot et al., 2019). In haemocytes, the percentage of cells containing autophagosomes significantly increased in oysters exposed to NH_4_Cl at 24 hpe (Picot et al., 2019). In the mantle, accumulation of the MAP1LC3-II protein (another key protein of the autophagy pathway) revealed inhibition of autophagy at 20 hpe as determined by western blotting (Moreau et al., 2015). The results of our experiment suggest that in *C. gigas*, expression of autophagy genes and proteins can be induced earlier than previously reported in the literature in the presence of NH_4_Cl.

The autophagy modulation was also investigated in oysters injected with OsHV-1. Several key genes of the autophagy pathway were significantly upregulated at 14 and 24–30 hpi in the mantle, and at late time points (18 to 30 hpi) of the experimental infection, in haemolymph. These results suggest that the virus can induce a modulation of autophagy genes. Upregulation of several *ATG* genes has already been reported at the transcriptional level after influenza virus infection (Klionsky et al., 2016). In the mantle of low-susceptibility Pacific oysters injected with OsHV-1, upregulation of *BECN1* at 8 to 12 hpi was reported (Moreau et al., 2015). In an analysis at different time points, we demonstrated here that several autophagy genes were upregulated at two time points in the mantle, whereas in the haemolymph, they were upregulated at one time point. These results point to different modulation of autophagy at the transcriptional level in the two tissues. Moreover, at the protein level, the expression of SQSTM1 significantly increased between 18 and 30 hpi in the mantle. In haemolymph, the percentage of cells containing autophagosomes significantly increased from 18 to 30 hpi. These results confirmed that OsHV-1 induced a modulation of the autophagy flux in the two tissues tested. In the mantle of *C. gigas*, a similar result was obtained by Moreau et al. (2015). Accumulation of the MAP1LC3-II protein was also demonstrated by western blotting, indicating that the autophagy flux was modulated at 20 hpi by OsHV-1. These findings are in agreement with the existing literature. For instance, induction of autophagy flux during the Sindbis virus infection in mouse embryonic fibroblasts was reported (Orvedahl et al., 2010; Chiramel et al., 2013).

In parallel, the viral replication was monitored. Viral DNA and RNA were detected in the two tissues starting from 6 hpi, indicating early replication of the virus in Pacific oysters. It has already been demonstrated that viral DNA can be detected in the mantle and the haemolymph of Pacific oyster spat since 6 hpi (Schikorski et al., 2011a). Moreover, viral transcripts of some OsHV-1 ORFs can be detected starting from 2 hpi in the mantle and from 1 h post contact in the haemolymph (Segarra et al., 2014b; Morga et al., 2017). By contrast, the viral replication kinetic seems to be different depending on the tissue considered. In haemolymph, the amounts of viral DNA and RNA strongly increased and reached a plateau, whereas in the mantle, the amount of viral DNA increased, and the expression levels of viral genes were stable. All our results indicate that the virus seems to start to replicate in the two tissues of *C. gigas* before the autophagy flux is modulated. Nonetheless, the autophagy modulation and the virus response are different between haemolymph and the mantle. It could be hypothesized that the two compartments regulate autophagy differently due to their different physiological functions. Dissimilar modulation of autophagy across different tissues of *Caenorhabditis elegans* in response to stress (starvation or anoxia) or aging has already been observed (Chapin et al., 2015). Moreover, it is also possible that in our study, the virus did not target the two compartments with the same aim and strategy. In fact, the mantle of *C. gigas* is an organ targeted by the virus, whereas in haemolymph, the haemocytes can serve as the cells transporting the virus OsHV-1 to target organs (Segarra et al., 2016; Morga et al., 2017).

Next, the autophagy modulation was followed in Pacific oysters exposed to NH_4_Cl and injected with the virus. In haemolymph and the mantle, autophagy gene expression levels increased starting from 6 hpi. In the presence of the virus and NH_4_Cl, this result means early modulation of autophagy genes in the two tissues. Moreover, the expression of protein SQSTM1 increased earlier and more strongly. The SQSTM1 protein quantity was found to be significantly higher in the virus+NH_4_Cl group than in the artificial seawater group at early (6 to 14 hpi) and late time points (18 to 30 hpi) of the experimental infection. In haemolymph, the percentage of cells containing autophagosomes significantly increased in the virus+NH_4_Cl group relative to the artificial seawater group at later time points of the experiment (18 to 30 hpi). In the two tissues, there was earlier and/or stronger induction of autophagy flux in the presence of the virus and NH_4_Cl than in the presence of the virus alone. This earlier and stronger autophagy induction can be partially due to the inhibition of autophagy by NH_4_Cl and an interaction with the infection process. It is important to remember that NH_4_Cl acts quite late in the autophagy process, inhibiting degradation of autophagosomes and promoting autophagosome accumulation. Moreover, autophagy is a process that is involved in the response to viral infections. This process can exert an antiviral action by degrading viral particles or viral proteins *via* its cellular mechanism (Tallóczy et al., 2006; Orvedahl et al., 2010; Judith et al., 2013; Sagnier et al., 2015). Nevertheless, it is known that the autophagy mechanism can be hijacked by viruses, e.g., herpesvirus among others, to enhance their replication, to be transported, or to exit the cell (Cavignac and Esclatine, 2010; Miszczak and Cymerys, 2014; Jackson, 2015; Lussignol and Esclatine, 2017).

In the mantle and haemolymph, no significant difference in viral DNA and RNA was detected between virus and virus+NH_4_Cl groups. In another study carried out at 20 hpi, no significant difference in viral DNA was observed in the mantle of Pacific oysters exposed to the virus and to virus+NH_4_Cl (Moreau et al., 2015). Unexpectedly, in these tissues, stronger and earlier modulation of autophagy does not induce a change in virus development. On the other hand, Moreau et al. (2015) demonstrated that inhibition of the autophagy pathway by NH_4_Cl increases the rate of mortality during OsHV-1 infection. Two hypotheses can be proposed to explain these results. Because the oyster family used here manifested an intermediate level of susceptibility to viral infection, it is possible that at the individual level, oysters can present a high level of variability in the severity of infection associated with high variability of viral DNA and RNA. This variability can conceal the effect of autophagy inhibition on viral replication. Besides, these results can suggest that inhibition of the degradation of autophagosomes by lysosomes has no effect on viral development in the two tissues. It is possible that the use of NH_4_Cl does not allow us to determine the role played by autophagy in the response to a viral infection. Perhaps the reason is that this compound does not inhibit the autophagic sequestration step to work.

Nevertheless, using other known inhibitors of the autophagy pathway to block autophagosome formation could be an alternative strategy to study the role play by autophagy during a viral infection. Several pharmacological agents are available to inhibit the nucleation step of autophagy (Galluzzi et al., 2017). Wortmannin and 3-methyladenine, two inhibitors of PIK3C3 function (class III phosphatidylinositol-3-kinase) are good candidates (Toogood, 2002; Rubinsztein et al., 2007). Nevertheless, the majority of these pharmacological agents serving to modulate the autophagy pathway have low pharmacological specificity for their target and can influence several other cellular pathways as well (Klionsky et al., 2016; Galluzzi et al., 2017). Therefore, in addition to the tested autophagy modulators, the role of autophagy in the response to viral infection needs to be investigated *via* other approaches (Klionsky et al., 2016).

## Conclusion

**Figure 6:**
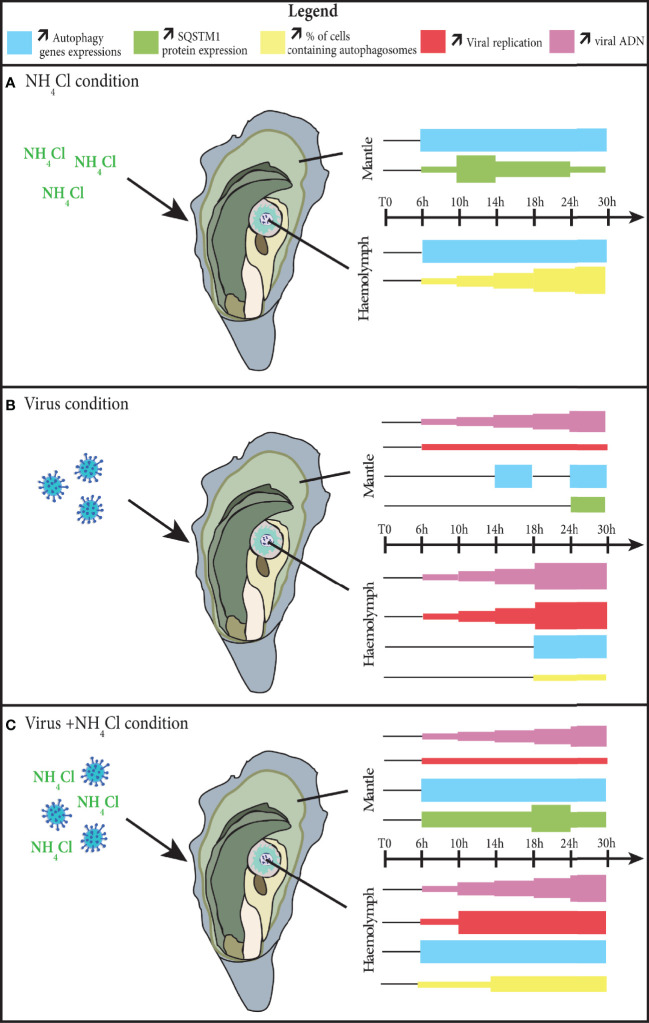
Modulation of autophagy in the mantle and haemolymph of the *Crassostrea gigas*, during the process of an infection by the virus OsHV-1. **(A)** in the NH_4_Cl condition; **(B)** in the virus condition; **(C)** in the virus+NH_4_Cl condition. The thickness of each color square represents an increase of the parameter considered.

In this study, we demonstrated that autophagy is active during infection by OsHV-1. The results showed that viral replication was initiated before autophagy was activated (**Figure 6**). Nevertheless, the autophagy modulation differs depending on the tissue being considered. Using a known inhibitor of autophagy, called NH_4_Cl, we found that autophagy can be inhibited beforehand in two tissues, the mantle and haemolymph, during the experiment. Because of the additive effects of NH_4_Cl and OsHV-1, earlier and stronger inhibition of autophagy was observed during the viral infection. Nevertheless, in the two tissues, inhibition of autophagy does not seem to be related to viral replication. Further research is needed to determine whether autophagy has an antiviral function or is manipulated by the virus for its own benefit.

## Data Availability Statement

The original contributions presented in the study are included in the article/**Supplementary Material**. Further inquiries can be directed to the corresponding author.

## Author Contributions

This study is the result of a collective work. SP, NF, and BM conceived this study and participated in its design. SP and NF performed the sample preparation for cellular and molecular analysis. SP, BM, NF, LD, IA, BC, and TR interpreted the results. SP and BM drafted the manuscript. All authors contributed to the article and approved the submitted version.

## Funding

This work received financial support from the European project VIVALDI (H2020 n°678589).

## Conflict of Interest

The authors declare that the research was conducted in the absence of any commercial or financial relationships that could be construed as a potential conflict of interest.

## Publisher’s Note

All claims expressed in this article are solely those of the authors and do not necessarily represent those of their affiliated organizations, or those of the publisher, the editors and the reviewers. Any product that may be evaluated in this article, or claim that may be made by its manufacturer, is not guaranteed or endorsed by the publisher.

## References

[B1] Allam B. Raftos D. (2015). Immune Responses to Infectious Diseases in Bivalves. J. Invertebr Pathol. 131, 121–136. doi: 10.1016/j.jip.2015.05.005 26003824

[B2] Azéma P. Lamy J.-B. Boudry P. Renault T. Travers M.-A. Dégremont L. (2017). Genetic Parameters of Resistance to *Vibrio Aestuarianus*, and Oshv-1 Infections in the Pacific Oyster, *Crassostrea Gigas*, at Three Different Life Stages. Genet. Sel. Evol. 49, 1–16. doi: 10.1186/s12711-017-0297-2 28201985 PMC5311879

[B3] Bachère E. Hervio D. Mialhe E. Grizel H. (1990). Evidence of Neutralizing Activity Against T3 Coliphage in Oyster *Crassostrea Gigas* Hemolymph. Dev. Comp. Immunol. 14, 261–268. doi: 10.1016/0145-305X(90)90017-9 2210005

[B4] Barbosa Solomieu V. Renault T. Travers M.-A. (2015). Mass Mortality in Bivalves and the Intricate Case of the Pacific Oyster, *Crassostrea Gigas* . J. Invertebr Pathol. 131, 2–10. doi: 10.1016/j.jip.2015.07.011 26210497

[B5] Berthelin C. Kellner K. Mathieu M. (2000). Storage Metabolism in the Pacific Oyster (*Crassostrea Gigas*) in Relation to Summer Mortalities and Reproductive Cycle (West Coast of France). Comp. Biochem. Physiol. Part B: Biochem. Mol. Biol. 125, 359–369. doi: 10.1016/S0305-0491(99)00187-X 10818269

[B6] Bjørkøy G. Lamark T. Pankiv S. Øvervatn A. Brech A. Johansen T. (2009). Chapter 12 Monitoring Autophagic Degradation of P62/SQSTM1. In ‘Methods in Enzymology’. Autophagy Mamm. Systems Part B, 181–197. (Academic Press) doi: 10.1016/S0076-6879(08)03612-4 19200883

[B7] Cavignac Y. Esclatine A. (2010). Herpesviruses and Autophagy: Catch Me If You can! Viruses 2, 314–333. doi: 10.3390/v2010314 21994613 PMC3185561

[B8] Chapin H. C. Okada M. Merz A. J. Miller D. L. (2015). Tissue-Specific Autophagy Responses to Aging and Stress in *C. Elegans* . Aging 7 (452), 419–434. doi: 10.18632/aging.100765 26142908 PMC4505168

[B9] Cherr G. N. Friedman C. S. (1998). “Investigation of a Mass Mortality of Pacific Oysters, Crassostrea Gigas,” in Tomales Bay, California. California Sea Grant Report of Completed Projects 1994-1997No, vol. 167–172. (La Jolla, CA: R-044 California Sea Grand College System).

[B10] Chiramel A. I. Brady N. R. Bartenschlager R. (2013). Divergent Roles of Autophagy in Virus Infection. Cells 2, 83–104. doi: 10.3390/cells2010083 24709646 PMC3972664

[B11] Davison A. J. Trus B. L. Cheng N. Steven A. C. Watson M. S. Cunningham C.. (2005). A Novel Class of Herpesvirus With Bivalve Hosts. J. Gen. Virol. 86, 41–53. doi: 10.1099/vir.0.80382-0 15604430

[B12] Degremont L. Bedier E. Soletchnik P. Ropert M. Huvet A. Moal J.. (2005). Relative Importance of Family, Site, and Field Placement Timing on Survival, Growth, and Yield of Hatchery-Produced Pacific Oyster Spat (*Crassostrea Gigas*). Aquaculture 249, 213–229. doi: 10.1016/j.aquaculture.2005.03.046

[B13] Deretic V. (2006). Autophagy as an Immune Defense Mechanism. Curr. Opin. Immunol. 18, 375–382. doi: 10.1016/j.coi.2006.05.019 16782319

[B14] Enríquez-Díaz M. Pouvreau S. Chávez-Villalba J. Le Pennec M. (2008). Gametogenesis, Reproductive Investment, and Spawning Behavior of the Pacific Giant Oyster *Crassostrea Gigas*: Evidence of an Environment-Dependent Strategy. Aquac Int. 17, 491. doi: 10.1007/s10499-008-9219-1

[B15] FAO (2018a) Fisheries & Aquaculture - Collections Statistiques De La Pêche - Production Mondiale De L’aquaculture. Available at: http://www.fao.org/fishery/statistics/global-aquaculture-production/fr (Accessed 9 September 2018).

[B16] FAO (2018b) Fisheries & Aquaculture - Programme d’Information Sur Les Espèces Aquatiques Cultivées - Crassostrea Gigas (Thunberg, 1793). Available at: http://www.fao.org/fishery/culturedspecies/Crassostrea_gigas/fr (Accessed 19 September 2018).

[B17] Friedman C. S. Shamseldin A. Pallai M. C. Olin P. G. (1997). Summer Mortality and the Stress Response of the Pacific Oyster, *Crassostrea Gigas* Thunberg. J. Shellfish Res. 16.

[B18] Gagnaire B. (2005). Etude Des Effets De Polluants Sur Les Paramètres Hémocytaires De L’huître Creuse, Crassostrea Gigas - Interactions Entre Environnement, Mécanismes De Défense Et Maladies Infectieuses (La Rochelle: Université de la Rochelle).

[B19] Galluzzi L. Bravo-San Pedro J. M. Levine B. Green D. R. Kroemer G. (2017). Pharmacological Modulation of Autophagy: Therapeutic Potential and Persisting Obstacles. Nat. Rev. Drug Discov. 16, 487–511. doi: 10.1038/nrd.2017.22 28529316 PMC5713640

[B20] Green T. J. Montagnani C. (2013). Poly I:C Induces a Protective Antiviral Immune Response in the Pacific Oyster (*Crassostrea Gigas*) Against Subsequent Challenge With Ostreid Herpesvirus (Oshv-1 μvar). Fish Shellfish Immunol. 35, 382–388. doi: 10.1016/j.fsi.2013.04.051 23685009

[B21] Green T. J. Raftos D. Speck P. Montagnani C. (2015). Antiviral Immunity in Marine Molluscs. J. Gen. Virol. 96, 2471–2482. doi: 10.1099/jgv.0.000244 26297577

[B22] Green T. J. Robinson N. Chataway T. Benkendorff K. O’Connor W. Speck P. (2014). Evidence That the Major Hemolymph Protein of the Pacific Oyster, *Crassostrea Gigas*, has Antiviral Activity Against Herpesviruses. Antiviral Res. 110, 168–174. doi: 10.1016/j.antiviral.2014.08.010 25169112

[B23] He Y. Jouaux A. Ford S. E. Lelong C. Sourdaine P. Mathieu M.. (2015). Transcriptome Analysis Reveals Strong and Complex Antiviral Response in a Mollusc. Fish Shellfish Immunol. 46, 131–144. doi: 10.1016/j.fsi.2015.05.023 26004318

[B24] Hine M. Wesney B. Hay B. (1992). Herpesviruses Associated With Mortalities Among Hatchery-Reared Larval Pacific Oysters *Crassostrea-Gigas* . Dis. Aquat. Organisms - Dis. AQUAT ORG 12, 135–142. doi: 10.3354/dao012135

[B25] Jackson W. T. (2015). Viruses and the Autophagy Pathway. Virology 479–480, 479–480. doi: 10.1016/j.virol.2015.03.042 PMC5917100 25858140

[B26] Judith D. Mostowy S. Bourai M. Gangneux N. Lelek M. Lucas-Hourani M.. (2013). Species-Specific Impact of the Autophagy Machinery on Chikungunya Virus Infection. EMBO Rep. 14, 534–544. doi: 10.1038/embor.2013.51 23619093 PMC3674439

[B27] Klionsky D. J. Abdelmohsen K. Abe A. Abedin M. J. Abeliovich H. Acevedo Arozena A.. (2016). Guidelines for the Use and Interpretation of Assays for Monitoring Autophagy (3rd Edition). Autophagy 12, 1–222. doi: 10.1080/15548627.2015.1100356 Adams. 26799652 PMC4835977

[B28] Klionsky D. J. Emr S. D. (2000). Autophagy as a Regulated Pathway of Cellular Degradation. Sci. (New York N.Y.) 290, 1717–1721. doi: 10.1126/science.290.5497.1717 PMC2732363 11099404

[B29] Levine B. Deretic V. (2007). Unveiling the Roles of Autophagy in Innate and Adaptive Immunity. Nat. Rev. Immunol. 7, 767–777. doi: 10.1038/nri2161 17767194 PMC7097190

[B30] Li Y. Qin J. G. Abbott C. A. Li X. Benkendorff K. (2007). Synergistic Impacts of Heat Shock and Spawning on the Physiology and Immune Health of *Crassostrea Gigas*: An Explanation for Summer Mortality in Pacific Oysters. Am. J. Physiol. Regul Integr. Comp. Physiol. 293, R2353–R2362. doi: 10.1152/ajpregu.00463.2007 17898119

[B31] Lussignol M. Esclatine A. (2017). Herpesvirus and Autophagy: “All Right, Everybody be Cool, This Is a Robbery!”. Viruses 9(12), 372. doi: 10.3390/v9120372 PMC5744147 29207540

[B32] Lynch S. A. Carlsson J. Reilly A. O. Cotter E. (2012). And Culloty, sA Previously Undescribed Ostreid Herpes Virus 1 (Oshv-1) Genotype Detected in the Pacific Oyster, *Crassostrea Gigas*, in Ireland. C. Parasitology 139, 1526–1532. doi: 10.1017/S0031182012000881 23036593

[B33] Martenot C. Gervais O. Chollet B. Houssin M. Renault T. (2017). Haemocytes Collected From Experimentally Infected Pacific Oysters, *Crassostrea Gigas*: Detection of Ostreid Herpesvirus 1 DNA, RNA, and Proteins in Relation With Inhibition of Apoptosis. PloS One 12, e0177448. doi: 10.1371/journal.pone.0177448 28542284 PMC5436676

[B34] Miszczak D. Cymerys J. (2014). A Game of Survival: Herpesvirus Strategies of Autophagy Manipulation. Adv. Hyg Exp. Med. 68, 1406–1414. doi: 10.5604/17322693.1130653 25531704

[B35] Mizushima N. (2005). The Pleiotropic Role of Autophagy: From Protein Metabolism to Bactericide. Cell Death Differ 12 Suppl 2, 1535–1541. doi: 10.1038/sj.cdd.4401728 16247501

[B36] Moreau P. Moreau K. Segarra A. Tourbiez D. Travers M.-A. Rubinsztein D. C.. (2015). Autophagy Plays an Important Role in Protecting Pacific Oysters From Oshv-1 and *Vibrio Aestuarianus* Infections. Autophagy 11, 516–526. doi: 10.1080/15548627.2015.1017188 25714877 PMC4502751

[B37] Morga B. Faury N. Guesdon S. Chollet B. Renault T. (2017). Haemocytes From *Crassostrea Gigas* and Oshv-1: A Promising *In Vitro* System to Study Host/Virus Interactions. J. Invertebr. Pathol. 150, 45–53. doi: 10.1016/j.jip.2017.09.007 28911815

[B38] Nicolas J. L. Comps M. Cochennec N. (1992). Herpes-Like Virus Infecting Pacific-Oyster Larvae, *Crassostrea Gigas* . Bull. Eur. Assoc. Fish Pathol. (United Kingdom). Ifremer.

[B39] Olicard C. Renault T. Torhy C. Benmansour A. Bourgougnon N. (2005). Putative Antiviral Activity in Hemolymph From Adult Pacific Oysters, *Crassostrea Gigas* . Antiviral Res. 66, 147–152. doi: 10.1016/j.antiviral.2005.03.003 15885817

[B40] Orvedahl A. MacPherson S. Sumpter R. Tallóczy Z. Zou Z. Levine B. (2010). Autophagy Protects Against Sindbis Virus Infection of the Central Nervous System. Cell Host Microbe 7, 115–127. doi: 10.1016/j.chom.2010.01.007 20159618 PMC2860265

[B41] Pankiv S. Clausen T. H. Lamark T. Brech A. Bruun J.-A. Outzen H.. (2007). P62/SQSTM1 Binds Directly to Atg8/LC3 to Facilitate Degradation of Ubiquitinated Protein Aggregates by Autophagy. J. Biol. Chem. 282, 24131–24145. doi: 10.1074/jbc.M702824200 17580304

[B42] Peeler E. J. Allan Reese R. Cheslett D. L. Geoghegan F. Power A. Thrush M. A. (2012). Investigation of Mortality in Pacific Oysters Associated With Ostreid Herpesvirus-1 μvar in the Republic of Ireland in 2009. Prev. Veterinary Med. 105, 136–143. doi: 10.1016/j.prevetmed.2012.02.001 22398251

[B43] Pepin J. F. Riou A. Renault T. (2008). Rapid and Sensitive Detection of Ostreid Herpesvirus 1 in Oyster Samples by Real-Time PCR. J. Virol Methods 149, 269–276. doi: 10.1016/j.jviromet.2008.01.022 18342377

[B44] Pfaffl M. W. (2001). A New Mathematical Model for Relative Quantification in Real-Time RT-PCR. Nucleic Acids Res. 29, e45. doi: 10.1093/nar/29.9.e45 11328886 PMC55695

[B45] Picot S. Faury N. Arzul I. Chollet B. Renault T. Benjamin M. (2020). Identification of the Autophagy Pathway in a Mollusk Bivalve, *Crassostrea Gigas* . Autophagy 16 (11), 2017–2035. doi: 10.1080/15548627.2020.1713643 31965890 PMC7595595

[B46] Picot S. Morga B. Faury N. Chollet B. Dégremont L. Travers M.-A.. (2019). A Study of Autophagy in Haemocytes of the Pacific Oyster, *Crassostrea Gigas* . Autophagy. 15 (10), 1801–1809. doi: 10.1080/15548627.2019.1596490 30939979 PMC6735588

[B47] Renault T. Cochennec N. Le Deuff R.-M. Chollet B. (1994a). Herpes-Like Virus Infecting Japanese Oyster (*Crassostrea Gigas*) Spat. Bull. Eur. Assoc. Fish Pathol. 14, 64–66.

[B48] Renault T. Faury N. Barbosa-Solomieu V. Moreau K. (2011). Suppression Substractive Hybridisation (SSH) and Real Time PCR Reveal Differential Gene Expression in the Pacific Cupped Oyster, *Crassostrea Gigas*, Challenged With Ostreid Herpesvirus 1. Dev. Comp. Immunol. 35, 725–735. doi: 10.1016/j.dci.2011.02.004 21371503

[B49] Renault T. Le Deuff R.-M. Cochennec N. Maffart P. (1994b). Herpesviruses Associated With Mortalities Among Pacific Oyster, *Crassostrea Gigas*, in France-Comparative Study. Rev. Médecine Vétérinaire 145, 735–742.

[B50] Renault T. Lipart C. Arzul I. (2001). A Herpes-Like Virus Infecting *Crassostrea Gigas* and *Ruditapes Philippinarum* Larvae in France. J. Fish Dis. 24, 369–376. doi: 10.1046/j.1365-2761.2001.00300.x

[B51] Rosani U. Varotto L. Domeneghetti S. Arcangeli G. Pallavicini A. Venier P. (2015). Dual Analysis of Host and Pathogen Transcriptomes in Ostreid Herpesvirus 1-Positive *Crassostrea Gigas* . Environ. Microbiol. 17, 4200–4212. doi: 10.1111/1462-2920.12706 25384719

[B52] Rubinsztein D. C. Gestwicki J. E. Murphy L. O. Klionsky D. J. (2007). Potential Therapeutic Applications of Autophagy. Nat. Rev. Drug Discov 6, 304–312. doi: 10.1038/nrd2272 17396135

[B53] Sagnier S. Daussy C. F. Borel S. Robert-Hebmann V. Faure M. Blanchet F. P.. (2015). Autophagy Restricts HIV-1 Infection by Selectively Degrading Tat in CD4+ T Lymphocytes. J. Virol. 89, 615–625. doi: 10.1128/JVI.02174-14 25339774 PMC4301118

[B54] Schikorski D. Faury N. Pepin J. F. Saulnier D. Tourbiez D. Renault T. (2011a). Experimental Ostreid Herpesvirus 1 Infection of the Pacific Oyster *Crassostrea Gigas*: Kinetics of Virus DNA Detection by Q-PCR in Seawater and in Oyster Samples. Virus Res. 155, 28–34. doi: 10.1016/j.virusres.2010.07.031 20709119

[B55] Schikorski D. Renault T. Saulnier D. Faury N. Moreau P. Pépin J.-F. (2011b). Experimental Infection of Pacific Oyster *Crassostrea Gigas* Spat by Ostreid Herpesvirus 1: Demonstration of Oyster Spat Susceptibility. Veterinary Res. 42, 27. doi: 10.1186/1297-9716-42-27 PMC3042938 21314910

[B56] Schmid D. Münz C. (2007). Innate and Adaptive Immunity Through Autophagy. Immunity 27, 11–21. doi: 10.1016/j.immuni.2007.07.004 17663981 PMC7118777

[B57] Segarra A. Baillon L. Faury N. Tourbiez D. Renault T. (2016). Detection and Distribution of Ostreid Herpesvirus 1 in Experimentally Infected Pacific Oyster Spat. J. Invertebr Pathol. 133, 59–65. doi: 10.1016/j.jip.2015.11.013 26674009

[B58] Segarra A. Baillon L. Tourbiez D. Benabdelmouna A. Faury N. Bourgougnon N.. (2014a). Ostreid Herpesvirus Type 1 Replication and Host Response in Adult Pacific Oysters, *Crassostrea Gigas* . Veterinary Res. 45, 103. doi: 10.1186/s13567-014-0103-x PMC4198667 25294338

[B59] Segarra A. Faury N. Pépin J.-F. Renault T. (2014b). Transcriptomic Study of 39 Ostreid Herpesvirus 1 Genes During an Experimental Infection. J. Invertebr Pathol. 119, 5–11. doi: 10.1016/j.jip.2014.03.002 24681357

[B60] Segarra A. Mauduit F. Faury N. Trancart S. Dégremont L. Tourbiez D.. (2014c). Dual Transcriptomics of Virus-Host Interactions: Comparing Two Pacific Oyster Families Presenting Contrasted Susceptibility to Ostreid Herpesvirus 1. BMC Genomics 15, 580. doi: 10.1186/1471-2164-15-580 25012085 PMC4111845

[B61] Segarra A. Pépin J. F. Arzul I. Morga B. Faury N. Renault T. (2010). Detection and Description of a Particular Ostreid Herpesvirus 1 Genotype Associated With Massive Mortality Outbreaks of Pacific Oysters, *Crassostrea Gigas*, in France in 2008. Virus Res. 153, 92–99. doi: 10.1016/j.virusres.2010.07.011 20638433

[B62] Sharifi M. N. Mowers E. E. Drake L. E. Macleod K. F. (2015). Measuring Autophagy in Stressed Cells. Methods Mol. Biol. (Clifton N.J.) 1292, 129–150. doi: 10.1007/978-1-4939-2522-3_10 PMC4460991 25804753

[B63] Tallóczy Z. Virgin H. W. Levine B. (2006). PKR-Dependent Autophagic Degradation of Herpes Simplex Virus Type 1. Autophagy 2, 24–29. doi: 10.4161/auto.2176 16874088

[B64] Toogood P. L. (2002). Inhibition of Protein-Protein Association by Small Molecules: Approaches and Progress. J. Medicinal Chem. 45, 1543–1558. doi: 10.1021/jm010468s 11931608

[B65] Webb S. C. Fidler A. Renault T. (2007)Primers for PCR-Based Detection of Ostreid Herpes Virus-1 (Oshv-1): Application in a Survey of New Zealand Molluscs (Accessed 13 November 2018).

[B66] Zhang G. Fang X. Guo X. Li L. Luo R. Xu F.. (2012). The Oyster Genome Reveals Stress Adaptation and Complexity of Shell Formation. Nature 490, 49–54. doi: 10.1038/nature11413 22992520

[B67] Zhang L. Li L. Zhang G. (2011). Gene Discovery, Comparative Analysis and Expression Profile Reveal the Complexity of the *Crassostrea Gigas* Apoptosis System. Dev. Comp. Immunol. 35, 603–610. doi: 10.1016/j.dci.2011.01.005 21237195

